# Development and preliminary evaluation toward a new tuberculosis treatment monitoring tool: the PATHFAST TB LAM Ag assay

**DOI:** 10.1128/jcm.00629-24

**Published:** 2024-07-19

**Authors:** Ayumi Akinaga, Masahito Takahashi, Takahito Yamazaki, Kinuyo Chikamatsu, Shuhei Matsushita, Yuichiro Hashimoto, Takako Iyoda, Takeshi Saika, Satoshi Mitarai

**Affiliations:** 1 PHC Corporation, Tokyo, Japan; 2 Department of Mycobacterium Reference and Research, The Research Institute of Tuberculosis, Japan Anti-Tuberculosis Association, Tokyo, Japan; 3 LSI Medience Corporation, Tokyo, Japan; University of Manitoba, Winnipeg, Manitoba, Canada

**Keywords:** lipoarabinomannan, tuberculosis, biomarker, bacterial load, immunoassay

## Abstract

The PATHFAST TB LAM Ag assay is based on a chemiluminescent enzyme immunoassay to quantify lipoarabinomannan (LAM) in sputum within 1 h, and was developed as an alternative to conventional culture methods for monitoring tuberculosis (TB) treatment. This study aimed to evaluate the analytical performance and initial clinical feasibility of using five *Mycobacterium tuberculosis* variants, 178 non-tuberculous mycobacteria (NTM), 34 upper respiratory and oral cavity microorganisms, 100 sputum specimens from untreated patients, and potential interfering substances, including 27 drugs. The results reveled a single-site repeatability coefficient of variation (CV) of 5.2%–7.0%, and a multi-site reproducibility CV of 7.1%–8.4%. The limit of blank, limit of detection, and limit of quantification were 3.03 pg/mL, 6.67 pg/mL, and 7.44 pg/mL, respectively. Linearity was observed over the analytical measurement range (10.0 pg/mL–50,000 pg/mL), and no hook effect was observed. The assay tended to cross-react with slow-growing NTMs, but not with common upper respiratory and oral cavity microorganisms, except *Nocardia asteroides*, *Nocardia farcinica*, and *Tsukamurella paurometabola*. No interference was observed in the presence of mucin, blood, or major anti-TB, anti-HIV, and anti-pneumonia drugs. Regarding clinical performance, the assay had a sensitivity of 88.8% (95% CI: 80.0%–94.0%) and specificity of 100.0% (95% CI: 83.9%–100.0%) using mycobacterial culture as the reference standard, and a correlation (Spearman’s *r* = −0.770) was observed between LAM concentration and time to detection of culture. These findings show, for the first time, that the PATHFAST TB LAM Ag assay has potential value for monitoring TB treatment.

## INTRODUCTION

Tuberculosis (TB), caused by *Mycobacterium tuberculosis* variants (MTB), remains a major global public health problem. In 2022, the World Health Organization (WHO) estimated 10.6 million new TB cases and 1.3 million TB-related deaths, with more than 80% occurring in low- and middle-income countries (LMICs) (1). Ending the global TB epidemic requires enhancing universal access to rapid and accurate diagnostics along with successful treatment (2).

For successful treatment, the absence of suitable assays to effectively identify treatment efficacy and optimize treatment poses a significant challenge (3). Conventional laboratory techniques, including smear microscopy (SM) and mycobacterial culture (4–7), suffer from limitations that hinder the prompt and accurate evaluation of treatment efficacy, especially for drug-resistant TB in LMICs (8, 9).

Despite its widespread use owing to its short turnaround time and low cost, SM lacks sensitivity and accuracy (10–13). Similarly, culture methods, which are considered the gold standard for monitoring TB treatment, are time-consuming, require specialized laboratory facilities and skilled personnel, carry the risk of contamination (14), and have higher occupational hazards related to TB (15, 16). Moreover, biosafety-adapted facilities are expensive, which limits access in many resource-limited settings. Consequently, novel technologies are required to address these deficiencies in monitoring TB treatment. Bacteriological biomarkers such as lipoarabinomannan (LAM) have emerged as potential candidates in this pursuit (3, 17, 18).

LAM, a glycolipid and major component of the mycobacterial cell wall, has been most studied as an MTB biomarker and has been detected in various biological samples, including sputum, urine, and blood (19–27). For urinary LAM, lateral flow assays such as Determine TB LAM Ag (Abbott, Chicago, IL, USA) and SILVAMP TB LAM Assay (Fujifilm, Tokyo, Japan) focus on detecting active TB in people living with HIV (23–25). Regarding sputum LAM, the TB LAM ELISA “Otsuka” (LAM-ELISA; Otsuka Pharmaceutical, Tokyo, Japan) has been evaluated in the development of anti-TB drug regimens. The LAM-ELISA has shown that the LAM concentration correlates with bacterial load and reflects the treatment response in patients with pulmonary TB during treatment (21, 22). While the LAM-ELISA provides results faster than the culture methods (within 5 h), it still faces challenges in achieving shorter measurement times, wider dynamic range, simpler operation, and correlation with bacterial load as treatment progresses.

To improve the feasibility of TB treatment monitoring, we at PHC Corporation (formerly LSI Medience Corporation, Tokyo, Japan) have developed the PATHFAST TB LAM Ag assay. This simple and automated chemiluminescent enzyme immunoassay (CLEIA) quantifies LAM in sputum within 1 h, including manual sample pretreatment. In this study, we evaluated the fundamental analytical and clinical performance, including the precision, detection limit, sensitivity, and specificity of the PATHFAST TB LAM Ag assay.

## MATERIALS AND METHODS

### Diagnostic system

#### PATHFAST TB LAM Ag assay

**Fig 1:**
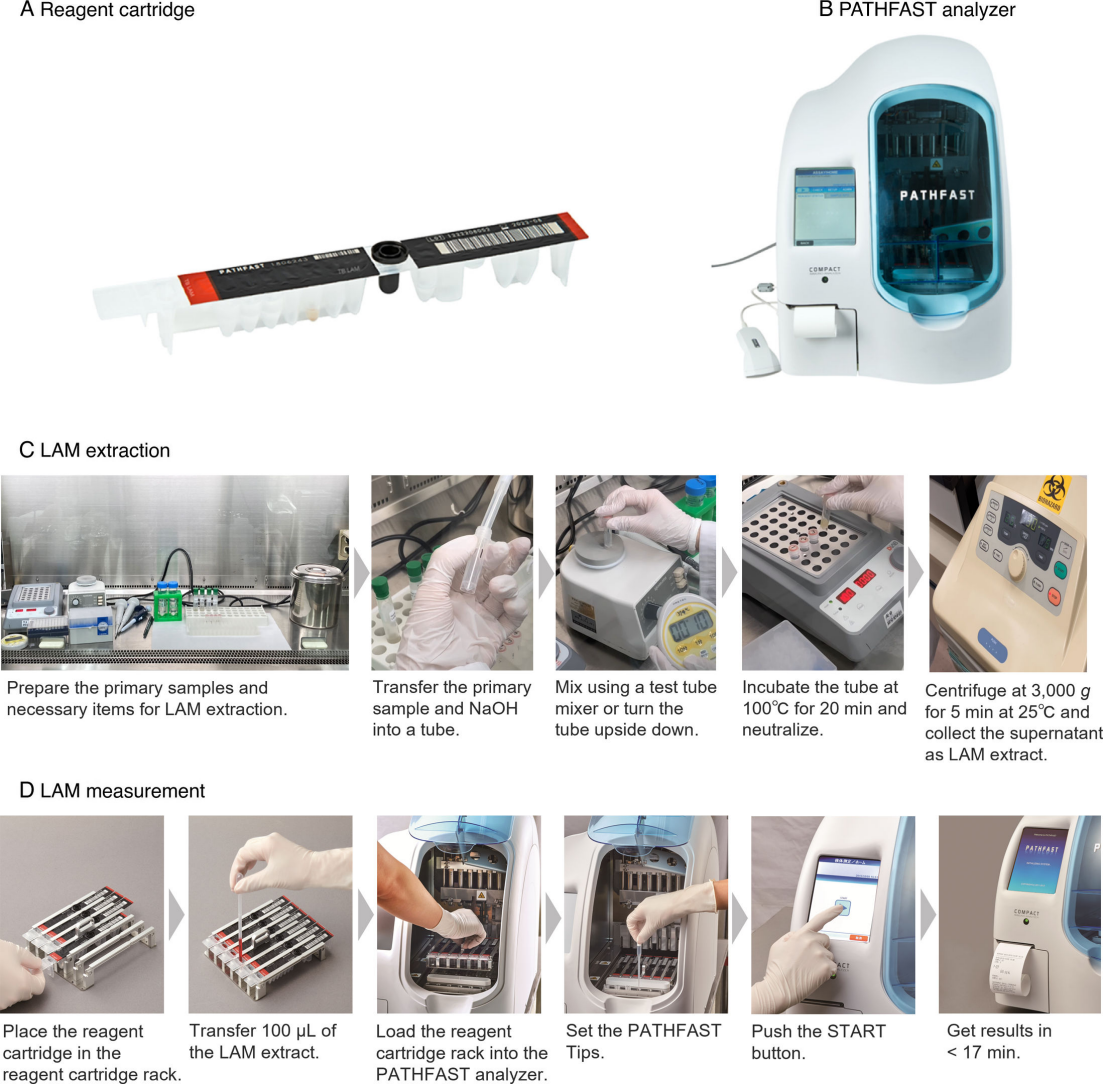
Overview of the PATHFAST TB LAM Ag assay and its operation flow. (**A**) The reagent cartridge is single use and filled with all necessary reagent components. (**B**) The PATHFAST analyzer is a bench-top analyzer based on chemiluminescent enzyme immunoassay, which does not require water and allows for up to six simultaneous tests with simple operation. (**C**) A minimum primary sample volume of 200 µL is required. Heating the sample with NaOH exposes the LAM antigen from bacilli, liquifies and decontaminates the sample, and inactivates the microorganism. (**D**) The PATHFAST analyzer automatically reads the barcode on the reagent cartridge and proceeds to analyze it.

PATHFAST TB LAM Ag [PHC Corporation (formerly LSI Medience Corporation), Tokyo, Japan] is a ready-to-use cartridge-based reagent for LAM quantification based on CLEIA and MAGTRATION technology. In brief, the assay is a two-step process consisting of (i) LAM extraction, which is performed manually, and (ii) LAM measurement, which is fully automated using a PATHFAST analyzer [PHC Corporation (formerly LSI Medience Corporation), Tokyo, Japan], as shown in Fig. 1. In the design of the PATHFAST TB LAM Ag assay, both the LAM extraction process and combination of antibodies in the LAM measurement process were developed based on Kawasaki’s method (21). The calibrator reference material for the PATHFAST TB LAM Ag assay was purified Bacille de Calmette et Guérin (BCG)-derived LAM and its concentration was determined using the LAM-ELISA.

In the LAM extraction process, 200 µL of sputum specimens or 800 µL of cultured microorganism suspensions were used as primary samples. Sputum specimens were carefully collected using a positive displacement pipette (MICROMAN E, Gilson, Wisconsin, USA) because of its viscous nature. A half volume of 1.0 N NaOH (FUJIFILM Wako Pure Chemical Corporation, Osaka, Japan) was added to each volume of the primary sample, before incubating at 100°C for 20 min. After incubation, the primary sample-NaOH mixture was neutralized with one-fourth volume of 5 M NaH_2_PO_4_ solution (Sigma-Aldrich Japan Inc., Tokyo, Japan), centrifuged at 3,000 *g* for 5 min at 25°C [or room temperature (RT)]. The supernatant was then gently collected as the LAM extract.

In the LAM measurement process, 100 µL of the LAM extract was transferred into the reagent cartridge, which was then loaded into the PATHFAST analyzer. Next, the sample was automatically mixed with magnetic particles coated with anti-LAM monoclonal antibodies (MoAbs; clones S4-20 and G3) and alkaline phosphatase (ALP)-labeled anti-LAM MoAb (clone TB). The immunocomplex was separated from the unbound ALP-labeled anti-LAM MoAb using a magnet, before adding CDP-Star and detecting luminescence. Finally, the LAM concentration was calculated within 17 min of the start of the assay using a standard curve.

### Samples

#### Clinical specimens

A total of 100 frozen raw sputum specimens from different patients were obtained from the Foundation for Innovative New Diagnostics (FIND, Geneva, Switzerland) biobank. Among these specimens, 80 were selected from patients with TB prior to the initiation of anti-TB drug treatment. This subset comprised 20 samples each from patients with SM negative-culture positive (S–C+), SM scanty-culture positive (S±C+), SM 1+-culture positive (S1+C+), and SM 2+ or 3+-culture positive (S2+C+ or S3+C+). The remaining 20 samples were selected from non-TB patients with SM negative-culture negative (S–C–). The specimens examined at FIND’s collaborating laboratories were subjected to concentrated fluorescent acid-fast bacilli staining, SM, Löwenstein-Jensen, and Mycobacteria Growth Indicator Tube (MGIT, Becton Dickinson and Company, Franklin Lakes, USA) culture, and Xpert MTB/RIF (Cepheid Inc., Sunnyvale, CA, USA). The LAM concentration was determined at the RIT and PHC Corporation (formerly LSI Medience Corporation) laboratories using the PATHFAST TB LAM Ag assay and LAM-ELISA.

#### *Mycobacterium tuberculosis* panel

Five MTB variants, namely, *M. tuberculosis* var. *tuberculosis* (ATCC 27294), *Mycobacterium tuberculosis* var. *africanum* (ATCC 25420), *Mycobacterium tuberculosis* var. *bovis* (ATCC 19210), *Mycobacterium tuberculosis* var. *microti* (ATCC 19422), and *Mycobacterium tuberculosis* var. *pinipedii* (ATCC BAA-688) were obtained from the ATCC (Virginia, USA). Each MTB variant was cultured in Myco broth medium (Kyokuto Pharmaceutical Industrial Co., Ltd, Tokyo, Japan). To determine the CFUs, each MTB suspensions were cultured on Middlebrook 7H10 agar (Nippon Becton Dickinson and Company, Tokyo, Japan) supplemented with 10% Oleic Acid Albumin Dextrose Catalase (Nippon Becton Dickinson and Company, Tokyo, Japan). After determining the CFUs, 10-fold serial dilutions of MTB suspensions from 1.0 × 10^2^ CFU/mL to 1.0 × 10^6^ CFU/mL were prepared.

#### Analytical specificity panel

A total of 178 non-tuberculous mycobacteria (NTM) and 34 pathogens or microorganisms from the upper respiratory and oral cavities were obtained from the Japan Collection of Microorganisms (JCM, Ibaraki, Japan), NITE Biological Resource Center (NBRC, Tokyo, Japan), ATCC (Virginia, USA), and German Collection of Microorganisms and Cell Cultures GmbH (DSMZ, Braunschweig, Germany). NTMs were cultured in Myco broth medium and bacterial solutions/suspensions were prepared ensuring a 0.1 optical density (O.D.) at 600 nm. *Chlamydophila pneumoniae* strains were cultured in HEp-2 cells, and bacterial inclusion bodies were counted. The other microorganisms were cultured to McFarland 2.0, and the CFU were obtained.

### Laboratory procedures

#### Precision

The single- and multi-site precisions of the PATHFAST TB LAM Ag assay were evaluated according to the Clinical Laboratory Standards Institute (CLSI) guideline EP05-A3 (28). Single-site precision was evaluated using four concentrations of sputum pools spiked with BCG-derived LAM and two concentrations of quality control (QC) materials [PATHFAST TB LAM Ag Control, PHC Corporation (formerly LSI Medience Corporation), Tokyo, Japan]. The spiked samples were prepared by adding BCG-derived LAM, previously treated with LAM extraction in phosphate buffer, to LAM extracts obtained from LAM-negative sputum pools. The QC materials were prepared by adding two concentrations of purified BCG-derived LAM [PHC Corporation (formerly LSI Medience Corporation), Tokyo, Japan]. Each sample was measured in duplicate in each run, with two runs per day for 20 days, using one reagent lot and one PATHFAST analyzer. The multisite precision was evaluated using the PATHFAST TB LAM Ag Control. The QC materials at two concentration levels were measured in five replicates for 5 days at three sites using one reagent lot, three operators, and three PATHFAST analyzers.

#### Detection limit

The limit of blank (LoB), limit of detection (LoD), and limit of quantification (LoQ) of the PATHFAST TB LAM Ag assay were evaluated according to CLSI guideline EP17-A2 (29). According to the CLSI guideline EP17-A2, the LoB is the “highest measurement result that is likely to be observed for a blank sample”; the LoD is the “lowest concentration of analyte that can be consistently detected”; and the LoQ is the “lowest concentration that can be quantitatively determined with stated accuracy.” Four blank sputum pools were measured in five consecutive replicates on each of the 3 test days using two reagent lots on two PATHFAST analyzers, for a total of 120 observations. Four low-concentration sputum pools spiked with BCG-derived LAM were measured in three consecutive replicates on each of the 5 test days using two reagent lots on two PATHFAST analyzers for a total of 120 observations. The spiked samples were prepared by adding BCG-derived LAM, previously treated with LAM extraction in phosphate buffer, to LAM extracts obtained from LAM-negative sputum pools. The LoB was calculated as follows: mean_blank_ + 1.645 (SD_blank_). The LoQ was calculated as follows: LoB + 1.645 (SD_low concentration sputum pool_). The LoQ was determined using the concentration at which the precision profile had a coefficient of variation (CV) of ≤20%.

#### Dilution linearity and hook effect

The dilution linearity was evaluated using four sputum pools with different concentrations of BCG-derived LAM, ranging from above the lower limit of quantitation (10.0 pg/mL) to the upper limit of quantitation (50,000 pg/mL). Samples that were fivefold serially diluted using PATHFAST SAMPLE DILUENT 1 [PHC Corporation (formerly LSI Medience Corporation), Tokyo, Japan] were measured in triplicate, and the %recovery for the theoretical concentration was calculated. The hook effect was assessed using sputum pools spiked with purified BCG-derived LAM at approximately 10,000,000 pg/mL. The samples were serially diluted twofold using PATHFAST SAMPLE DILUENT 1 and measured in triplicate.

#### Potential interfering substances

The following materials were tested: 1,000 µg/mL mucin from porcine stomach, 10% (vol/vol) human blood, and 27 drugs at 100 µg/mL for TB, pneumonia, and HIV, including isoniazid, rifampicin, streptomycin sulfate, ethambutol, ethionamide, pyrazinamide, kanamycin sulfate, enviomycin sulfate, cycloserine, azithromycin, clarithromycin, cefditoren pivoxil, minocycline hydrochloride, imipenem-cilastatin sodium, levofloxacin, povidone-iodine, betamethasone sodium phosphate-fradiomycin sulfate, potassium clavulanate-amoxicillin hydrate, lamivudine, emtricitabine, abacavir sulfate, tenofovir disoproxil fumarate, efavirenz, etravirine, rilpivirine, atazanavir sulfate, and maraviroc. Each material was introduced into a phosphate buffer spiked with purified BCG-derived LAM.

#### LAM-ELISA

The TB LAM ELISA “Otsuka” kits were obtained from Otsuka Pharmaceutical Co. Ltd. (Tokyo, Japan). The reference material for the calibrator included in the kit was purified LAM from the MTB Aoyama B strain. The assay was performed according to the manufacturer’s instructions. In brief, 100 µL of LAM extracts were placed in the ELISA plate and incubated for 90 min at RT. After washing, a biotin-conjugated detection antibody was added to the ELISA plate and incubated for 90 min at RT. The plate was then washed again, and horseradish peroxidase-conjugated streptavidin was added, followed by incubation for 90 min at RT. After another wash, 3,3′,5′,5′-tetramethylbenzidine dihydrochloride and hydrogen peroxide were added, and the plate was incubated for 15 min at RT. Color development was stopped by the addition of sulfuric acid. The O.D. was measured at 450 nm using a microplate reader ELx808 (BioTek Instruments, Inc., Vermont, US). LAM concentration was calculated using a standard curve for each plate.

### Statistical analysis

The software “Analyze-it” (Analyze-it Software, Ltd., Leeds, UK) was employed for all statistical analyses. A significance level of *P* < 0.05 was used to determine statistical significance.

## RESULTS

### Precision

Table 1 shows the single-site precision using four sputum pools (sputum pools 1–4) and two QC materials (controls 1 and 2), covering the low-to-high analytical measurement range (10.0 pg/mL–50,000 pg/mL). The mean values for sputum pools 1 through 4 were 109 pg/mL, 2,564 pg/mL, 22,861 pg/mL, and 40,878 pg/mL, while the mean values for controls 1 and 2 were 92.6 pg/mL and 33,475 pg/mL, respectively. The repeatability CVs ranged from 5.2% to 7.0%, and the within-laboratory CVs ranged from 5.8% to 7.6% across the low-to-high-concentration ranges.

**TABLE 1:** Single-site precision performance^*a*^

Sample	Mean (pg/mL)	Repeatability		Within-laboratory	
		SD (pg/mL)	%CV (%)	SD (pg/mL)	%CV (%)
Sputum pool 1	109	6.47	5.9	6.57	6.0
Sputum pool 2	2,564	158	6.2	171	6.7
Sputum pool 3	22,861	1,292	5.7	1,322	5.8
Sputum pool 4	40,878	2,558	6.3	2,736	6.7
Control 1	92.6	6.48	7.0	7.01	7.6
Control 2	33,475	1,726	5.2	2,004	6.0

^*a*^ %CV, coefficient of variation expressed as a percentage.

Table 2 shows the multi-site precision for the three sites. The mean values for controls 1 and 2 were 96.2 pg/mL and 32,803 pg/mL, with repeatability CVs of 6.6% and 6.7%, within-site CVs of 7.0% and 7.7%, and reproducibility CVs of 7.1% and 8.4%, respectively.

**TABLE 2:** Multi-site precision performance across three sites^*a*^

Sample	Mean (pg/mL)	Repeatability		Within-site		Total (reproducibility)	
		SD (pg/mL)	%CV (%)	SD (pg/mL)	%CV (%)	SD (pg/mL)	%CV (%)
Control 1	96.2	6.38	6.6	6.74	7.0	6.82	7.1
Control 2	32,803	2,212	6.7	2,534	7.7	2,750	8.4

^*a*^ %CV, coefficient of variation expressed as a percentage.

### Detection limit

The detection limit was evaluated using sputum pools. The average blank value was 1.35 pg/mL with a standard deviation of 1.02 pg/mL, resulting in a calculated LoB of 3.03 pg/mL. In the case of the four LAM-spiked samples, the combined standard deviation was 2.21 pg/mL (Table S1), establishing the LoD at 6.67 pg/mL. Using the precision profile approach and 20% CV, the LoQ was determined to be 7.44 pg/mL.

### Dilution linearity and hook effect

Table 3 shows the dilution linearity using serially diluted sputum pools within the analytical measurement range of 10 pg/mL–50,000 pg/mL, which is wider than the 15 pg/mL–750 pg/mL range of LAM-ELISA. The %CV of the serially diluted sputum pools ranged from 1.3% to 10.1%, while that of all in-range samples was ≤20%. The %recovery ranged from 93.8% to 117.5%, while that of all in-range samples was within 100 ± 25% of the theoretical concentrations.

**TABLE 3:** Dilution linearity^*a*^

Sample	Dilution factor	Mean (pg/mL)	SD (pg/mL)	%CV (%)	%Recovery (%)
Very low	1/5	27.9	2.82	10.1	104.9
	2/5	55.6	0.74	1.3	104.5
	3/5	93.8	2.03	2.2	117.5
	4/5	114	5.51	4.8	107.1
	5/5	133	3.21	2.4	100.0
Low	1/5	749	34.8	4.6	111.7
	2/5	1,345	57.8	4.3	100.3
	3/5	1,990	82.5	4.1	98.9
	4/5	2,518	119	4.7	93.8
	5/5	3,354	157	4.7	100.0
Middle	1/5	6,116	237	3.9	101.1
	2/5	12,730	847	6.7	105.2
	3/5	17,737	1,203	6.8	97.7
	4/5	23,224	1,531	6.6	96.0
	5/5	30,247	987	3.3	100.0
High	1/5	8,754	703	8.0	96.2
	2/5	17,144	487	2.8	94.2
	3/5	28,193	1,781	6.3	103.2
	4/5	34,552	1,020	3.0	94.9
	5/5	45,514	1,666	3.7	100.0

^*a*^ %CV, coefficient of variation expressed as a percentage.

Furthermore, in samples where the LAM concentration exceeded the upper limit of measurement at 50,000 pg/mL and reached up to 10,411,349 pg/mL, the measurements were output as >50,000 pg/mL, and no hook effect causing false negatives was observed.

### Reactivity of type strain of MTB variants

**Fig 2:**
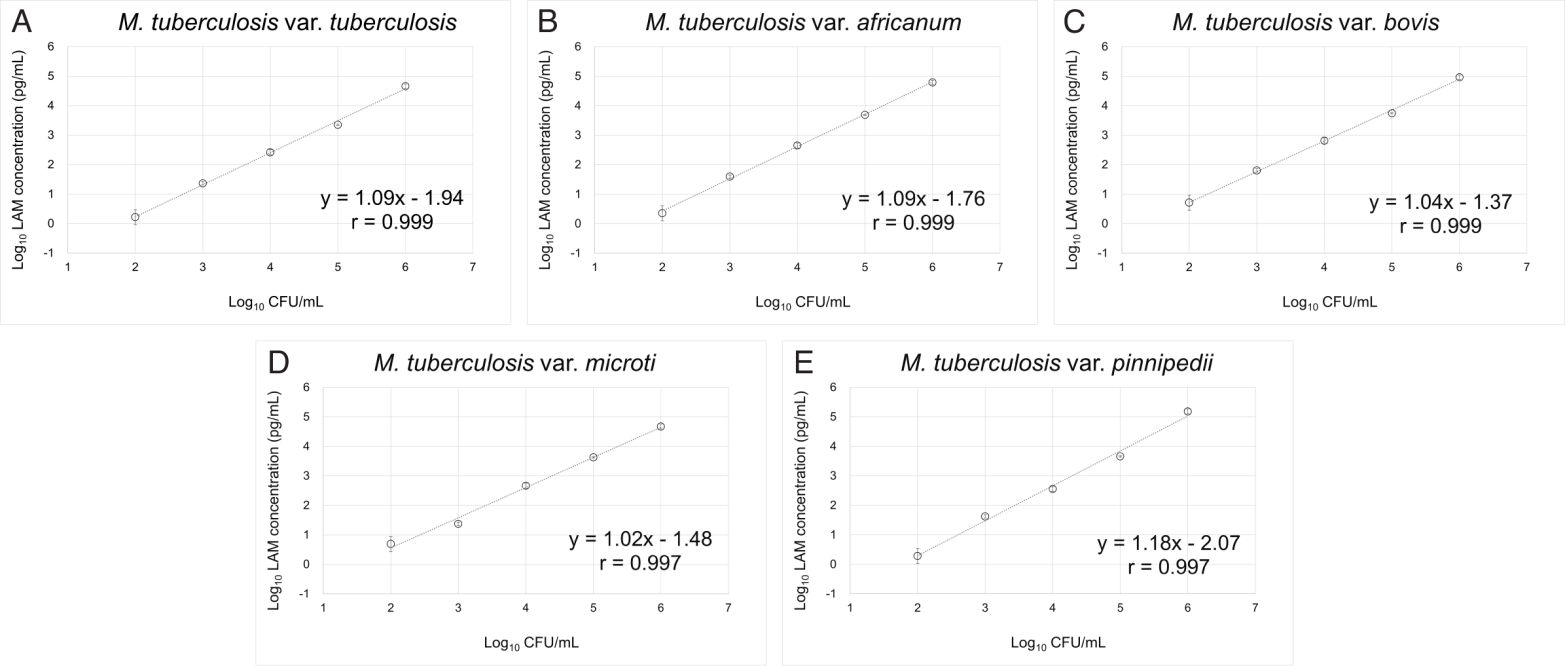
LAM concentrations ranging from 10^2^ to 10^6^ CFU/mL of MTB variants. Each bacterial suspension of 10^2^ to 10^6^ CFU/mL was prepared by 10-fold serial dilution in Middlebrook 7H9 broth with 0.05% Tween 80. These dilutions were used for LAM extraction, and the LAM concentrations were measured in triplicate. The mean value and standard deviation of the respective LAM concentrations at each bacterial suspension are plotted in the figure.

Figure 2 shows the reactivity against five MTB variants, including *M. tuberculosis* var. *tuberculosis*, *M. tuberculosis* var. *africanum*, *M. tuberculosis* var. *bovis*, *M. tuberculosis* var. *microti,* and *M. tuberculosis* var. *pinipedii*. For all MTB variants evaluated, the assay detected LAM, and a log-linear relationship was observed between the LAM concentration and CFU counts, ranging from 1.0 × 10^2^ CFU/mL to 1.0 × 10^6^ CFU/mL (*r* = 0.997–0.999). According to the calculated relationship between the LAM concentration and CFU count based on the regression equation, 1 pg/mL LAM corresponds to 50 CFU/mL of *M. tuberculosis* var. *tuberculosis* (Fig. 2A).

### Analytical specificity

The LAM concentrations were tested in 178 NTMs, including clinically important NTMs, obtained from an analysis of bacterial suspension at an O.D. of 0.01 (approximately 1.0 × 10^6^ CFU/mL). The assay tended to cross-react with slow-growing NTMs (79.1%, 68/86) such as *Mycobacterium avium*, *Mycobacterium intracellulare,* and *Mycobacterium kansasii* (Table S2A). In contrast, the assay did not cross-react with rapid-growing NTMs (85.9%, 79/92), including *Mycobacterium abscessus* (Table S2B).

Additionally, the LAM concentrations in 34 common upper respiratory tract pathogens and microorganisms in the oral cavity were examined (Table S3). *Chlamydophila pneumoniae* at 5.9 × 10^5^ cells/mL and other microbial suspensions at McFarland turbidity standard no. 2 (1.2 × 10^7^ to 1.9 × 10^9^ CFU/mL) were examined. The assay weakly cross-reacted with *Nocardia asteroides*, *Nocardia farcinica*, and *Tsukamurella paurometabola* but not with other microbial suspensions. The bacterial CFU numbers without cross-reactivity were 1.0 × 10^7^ CFU/mL for *N. asteroides*, 1.0 × 10^8^ CFU/mL for *N. farcinica*, and 1.0 × 10^8^ CFU/mL for *T. paurometabola*.

### Potential interfering substances

The potential for interference in the assay results was tested using final concentration of 1,000 µg/mL of mucin, 5% blood (vol/vol), and 27 drugs at 100 µg/mL for TB, HIV, and pneumonia (Table S4). None of the tested substances interfered with the assay performance.

**TABLE 4:** Clinical sensitivity and specificity of TB detection in sputum of pretreatment patients^*c*^

Assay	Sensitivity [*n*/*n* (%) (95% CI)]			Specificity [*n*/*n* (%) (95% CI)]	Overall agreement [*n*/*n* (%) (95% CI)]
	S+C+	S–C+	C+	C−	All
PATHFAST TB LAM Ag	59/60^*a*^ (98.3) (91.1–99.7)	12/20^*b*^ (60.0) (38.7–78.1)	71/80 (88.8) (80.0–94.0)	20/20 (100.0) (83.9–100.0)	91/100 (91.0) (83.8–95.2)
LAM-ELISA	57/60 (95.0) (86.3–98.3)	5/20 (25.0) (11.2–46.9)	62/80 (77.5) (67.2–85.3)	20/20 (100.0) (83.9–100.0)	82/100 (82.0) (73.3–88.3)
Xpert MTB/RIF	58/58 (100.0) (93.8–100.0)	14/20 (70.0) (48.1–85.5)	72/78 (92.3) (84.2–96.4)	20/20 (100.0) (83.9–100.0)	98/98 (93.9) (87.3–97.2)
Smear microscopy	–	–	60/80 (75.0) (64.5–83.2)	20/20 (100.0) (83.9–100.0)	80/100 (80.0) (71.1–86.7)

^*a*^ One sample that was negative on the PATHFAST TB LAM Ag assay was scant for smear microscopy, negative for LAM-ELISA, and positive for Xpert MTB/RIF.

^*b*^ Eight samples that were negative in the PATHFAST TB LAM Ag assay were also negative in the LAM-ELISA. Four of the eight samples that were negative in the PATHFAST TB LAM Ag assay were positive by Xpert MTB/RIF.

^*c*^ S, smear microscopy; C, culture. The culture methods were used as the reference standard.

### Clinical sensitivity and specificity for detecting TB

The clinical sensitivity and specificity were evaluated using raw sputum specimens from the FIND biobank. Specimens were collected from 80 untreated TB and 20 non-TB subjects from Vietnam and Peru. Males constituted the majority of cases (67; 67.0%), 14 (14.0%) were HIV-positive, and the median age was 37 years [interquartile range (IQR): 30.0–49.0].

A cutoff value of 10 pg/mL for the PATHFAST TB LAM Ag assay was determined as the point where Youden’s index was maximum on receiver operating characteristic analysis. A cutoff value of 15 pg/mL for the LAM-ELISA was selected based on Kawasaki’s study (21), and this point was the LoQ of the LAM-ELISA.

The PATHFAST TB LAM Ag assay showed a sensitivity of 88.8% (95% CI: 80.0%–94.0%), specificity of 100.0% (95% CI: 83.9%–100.0%), and overall agreement of 91.0% (95% CI: 83.8%–95.2%) (Table 4). Compared to the sensitivity, specificity, and overall agreement of the PATHFAST TB LAM Ag assay and other tests, significant differences (*P* < 0.05) were found between the sensitivity of the PATHFAST TB LAM Ag assay and SM and the overall agreement of the PATHFAST TB LAM Ag assay and SM.

### Relationship between LAM concentrations and bacterial load

To investigate whether the LAM concentration determined by the PATHFAST TB LAM Ag assay reflects the bacterial load in clinical sputum samples, the correlation between the LAM concentration and the SM score or time to detection (TTD) of MGIT was examined.

**Fig 3:**
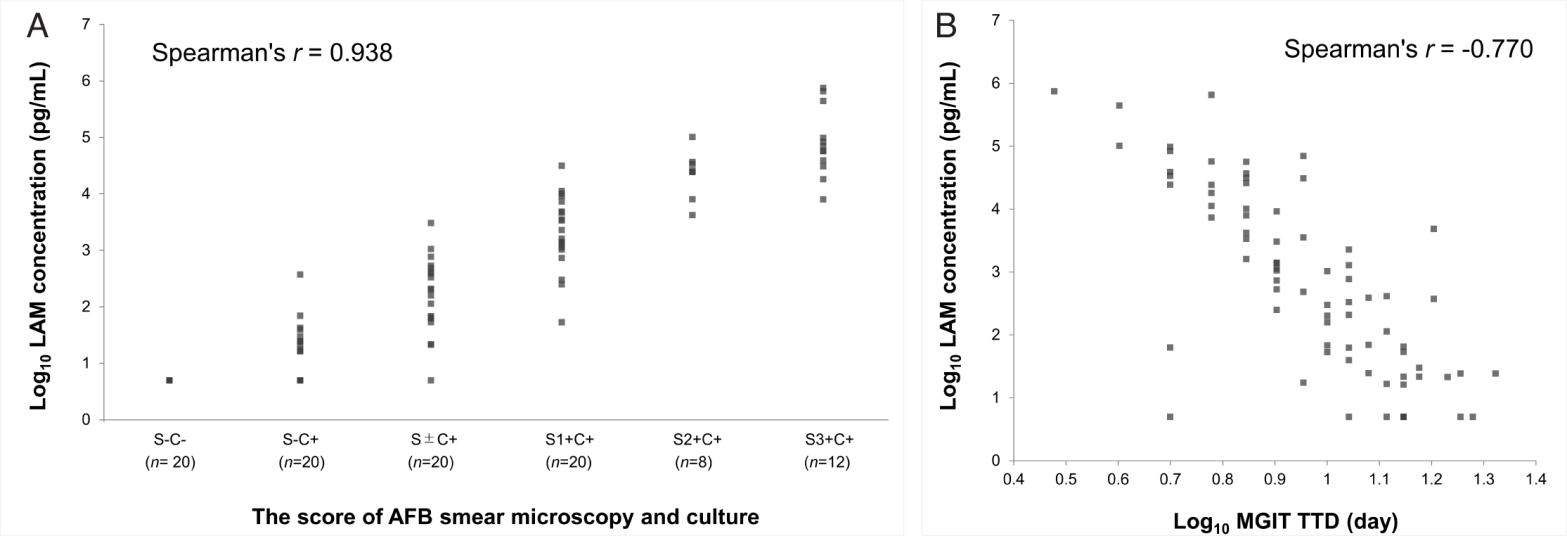
Relationship between LAM concentrations by the PATHFAST TB LAM Ag assay and bacterial load. (**A**) Correlation of LAM concentration by the PATHFAST TB LAM Ag assay with smear microscopy and culture scores. (**B**) Correlation of LAM concentration by the PATHFAST TB LAM Ag assay with TTD of MGIT. S, smear microscopy; C, culture.

In this study, the score of SM was combined results of culture, grading as SM negative-culture negative (S–C−), SM negative-culture positive (S–C+), SM scanty-culture positive (S±C+), SM 1+-culture positive (S1+C+), SM 2+-culture positive (S2+C+), and SM 3+-culture positive (S3+C+). The LAM concentration determined by the PATHFAST TB LAM Ag assay showed an upward trend depending on the SM and culture scores, with a Spearman’s rank correlation coefficient of 0.938 (95% CI: 0.909–0.959) (Fig. 3A). The correlation between the LAM concentration and MGIT TTD is shown in Fig. 3B. Of the 100 samples examined, 77 samples that were MGIT-positive and had TTD results were used for this evaluation. The LAM concentration correlated well with the MGIT TTD, with a Spearman’s rank correlation coefficient of −0.770 (95% CI: −0.849 to −0.655).

## DISCUSSION

One of the main goals of this study was to determine whether the PATHFAST TB LAM Ag assay exhibits the fundamental analytical and clinical performance necessary for practical and achievable TB treatment monitoring, and can serve as an alternative to conventional culture methods. Our results, encompassing the analytical precision, linearity, and analytical and clinical sensitivity and specificity of the assay, along with its operational and safety advantages, suggest its potential as a monitoring tool for patients with TB.

The PATHFAST TB LAM Ag assay is expected to provide rapid and straightforward measurements using a cartridge-based reagent and an automated analyzer (Fig. 1). The rapidity of less than 1 h is comparable to that of SM, and significantly outperforms culture methods, which take several days to 6–8 weeks, and the LAM-ELISA, which requires 5 h.

The LAM extraction process, although manual, is simpler than the decontamination with N-acetyl *L*-cysteine-NaOH used in culture methods. Culture methods are also susceptible to bacterial contamination even after decontamination, particularly in LMICs, where contamination rates can be significantly higher, reaching 15.7% in Kenya (30) and 28.2% in Sudan (31). In contrast, the PATHFAST TB LAM Ag assay avoids the loss of results and prolonged processing times.

Furthermore, the PATHFAST TB LAM Ag LAM potentially offers safety advantages over culture methods. The risk of infections is heightened in LMICs, where there is often lack of personal protective equipment and necessary training to prevent occupational respiratory infections (32). Studies by Doig (33) and Sabiiti (34) demonstrated that a heating process for 20 min at 80°C effectively inactivated mycobacteria. Thereby, the PATHFAST TB LAM Ag, which includes a heating process at 100°C for 20 min, has potential to reduce the risk to levels similar to those of SM.

In this analytical performance study, the PATHFAST TB LAM Ag assay yielded reproducible results at both single- and multi-site (Tables 1 and 2). Compared to the CFU counts required for the detection of mycobacteria, 10–100 CFU/mL for culture methods and 1,000–10,000 CFU/mL for SM (35), the LoQ of this assay was less than 10 pg/mL, corresponding to 500 CFU/mL of *M. tuberculosis* type strain (Fig. 2A). Additionally, the assay showed dilution linearity within the measurement range of 10 pg/mL–50,000 pg/mL (Table 3).

Several SM quality assessment studies in LMICs reported poor laboratory quality performance (7–10). Errors have been reported to vary widely, with rates ranging from 0% to 21% for false negative and from 0.1% to 19% for false positives (15). Technical gaps in smear preparation, staining, and reading procedures contribute to these errors (7–10, 15), which are often exacerbated by infrequent interventions or a lack of timely feedback (15). Therefore, the PATHFAST TB LAM Ag assay, which is simple, automated, and highly accurate, is expected to minimize operator error and enhance diagnostic reliability.

In this analytical specificity study, the PATHFAST TB LAM Ag assay showed a tendency to cross-react with NTMs belonging to slow-growing mycobacteria, to which MTB variants also belong (Table S2A and B), and weakly reacted with *N. asteroides*, *N. farcinica*, and *T. paurometabola* (Table S3). However, the assay showed no cross-reactivity with common upper respiratory tract pathogens or microorganisms found in the oral cavity (Table S3). This cross-reactivity trend was also consistent with that observed in Kawasaki’s study using the LAM-ELISA (21).

It is noteworthy that the PATHFAST TB LAM Ag assay has difficulty distinguishing between MTB and NTM, as in SM, and exhibits cross-reactivity with certain species of microorganisms. However, these disadvantages do not appear to be substantial when the assay is used as a monitoring tool for patients initially diagnosed with TB.

In this clinical evaluation using 100 biobank raw sputum samples, the PATHFAST TB LAM Ag assay showed 88.8% (71/80) sensitivity and 100.0% (20/20) specificity for detecting TB in pretreatment patients. While the assay did not detect all cases with SM-negative culture-positive samples, its sensitivity was comparable to that of the LAM-ELISA and Xpert MTB/RIF and better than that of SM (Table 4). The LAM concentration determined by the PATHFAST TB LAM Ag assay showed upward trends consistent with the SM and culture scores as well as the TTD of MGIT (Fig. 3).

Kawasaki (21) and Jones (22) reported that LAM concentration measured by the LAM-ELISA correlated well with the TTD of MGIT and bacterial CFU count, indicating that LAM is a pharmacodynamic biomarker for measuring the MTB burden. Although the sensitivity of the PATHFAST TB LAM Ag assay has limitations in detecting TB in patients with low bacterial loads, the results of this study suggest that LAM measured using the PATHFAST TB LAM Ag assay, similar to the LAM-ELISA, could serve as a potential biomarker for monitoring TB treatment.

This study revealed several advantages of the PATHFAST TB LAM Ag assay, such as straightforward measurement that minimizes differences in operator skill, as well as safety, accuracy, and high sensitivity. However, it is important to acknowledge the limitations of this study. Ongoing challenges remain regarding technical differences among operators owing to the manual nature of the LAM extraction process and the lack of methods to confirm successful LAM extraction. Additionally, the multi-site precision study was limited to only three facilities, and the clinical performance study included a relatively small sample size of untreated subjects.

To facilitate wider implementation in diverse clinical settings and solidify its role in TB treatment monitoring, it will be necessary to conduct further extensive validation across various patients with TB during different treatment periods by a range of users in different geographical regions. Moreover, to evaluate clinical performance, it is important to assess the reactivity of the assay with viable and dead bacilli at different time points over several months of treatment. Additionally, it is essential to evaluate the ability of the assay to identify patients who need intensive treatment or are at risk of poor outcomes during and after treatment, as required by the WHO (16).

In conclusion, this study presented for the first time that the PATHFAST TB LAM Ag assay rapidly and reproducibly quantified LAM with high sensitivity, and that the LAM concentration correlated well with the bacterial load. This assay holds promise for the improved clinical management of patients on therapy, potentially serving as an alternative to culture methods. Continued research and validation efforts are imperative to establish its reliability and suitability across diverse settings.

## SUPPLEMENTAL MATERIAL

Tables S1 to S4 jcm.00629-24-s0001.docx Measurements of LoD, cross-reactivity, and potential interfering substances.

ASM does not own the copyrights to Supplemental Material that may be linked to, or accessed through, an article. The authors have granted ASM a non-exclusive, world-wide license to publish the Supplemental Material files. Please contact the corresponding author directly for reuse.
