# Supplementary material for: Development and preliminary evaluation toward a new tuberculosis treatment monitoring tool: the PATHFAST TB LAM Ag assay
Source: J Clin Microbiol. 2024 Jul 19;62(8):e00629-24. doi: 10.1128/jcm.00629-24 (PMC11323533; doi:10.1128/jcm.00629-24)
Supplement: Tables S1 to S4 — Measurements of LoD, cross-reactivity, and potential interfering substances. [file jcm.00629-24-s0001.docx]

**Supplementary Table 1.** Measurements of LoD test samples

| Day | Reagent  Lot |  | LAM (pg/mL) | | | |
| --- | --- | --- | --- | --- | --- | --- |
|  |  |  | Sputum  Pool 1 | Sputum  Pool 2 | Sputum  Pool 3 | Sputum  Pool 4 |
| 1 | 1 | n=1 | 33.6 | 17.2 | 8.36 | 4.46 |
|  |  | n=2 | 39.1 | 16.0 | 9.47 | 4.59 |
|  |  | n=3 | 37.3 | 16.8 | 8.62 | 4.26 |
|  | 2 | n=1 | 32.7 | 14.3 | 6.99 | 3.04 |
|  |  | n=2 | 32.9 | 12.3 | 8.07 | 4.00 |
|  |  | n=3 | 30.1 | 12.7 | 7.17 | 2.88 |
| 2 | 1 | n=1 | 37.8 | 16.2 | 8.52 | 5.30 |
|  |  | n=2 | 39.7 | 16.8 | 9.67 | 4.33 |
|  |  | n=3 | 39.3 | 16.4 | 9.36 | 5.10 |
|  | 2 | n=1 | 30.4 | 16.8 | 6.88 | 1.50 |
|  |  | n=2 | 33.5 | 13.3 | 8.36 | 2.34 |
|  |  | n=3 | 31.2 | 14.2 | 6.46 | 3.64 |
| 3 | 1 | n=1 | 37.4 | 17.4 | 9.21 | 5.15 |
|  |  | n=2 | 37.5 | 20.0 | 8.67 | 6.48 |
|  |  | n=3 | 37.0 | 15.0 | 10.9 | 4.90 |
|  | 2 | n=1 | 34.6 | 14.8 | 8.31 | 3.27 |
|  |  | n=2 | 36.0 | 17.7 | 9.35 | 3.98 |
|  |  | n=3 | 34.8 | 15.9 | 8.82 | 5.05 |
| 4 | 1 | n=1 | 38.9 | 16.7 | 8.84 | 5.46 |
|  |  | n=2 | 42.0 | 16.8 | 10.7 | 5.72 |
|  |  | n=3 | 38.8 | 13.6 | 9.28 | 4.21 |
|  | 2 | n=1 | 34.2 | 14.9 | 5.83 | 4.92 |
|  |  | n=2 | 39.0 | 15.1 | 6.49 | 3.58 |
|  |  | n=3 | 32.6 | 13.9 | 5.94 | 3.95 |
| 5 | 1 | n=1 | 39.6 | 18.6 | 9.78 | 6.16 |
|  |  | n=2 | 43.5 | 16.9 | 11.6 | 5.39 |
|  |  | n=3 | 42.3 | 17.5 | 9.06 | 4.32 |
|  | 2 | n=1 | 33.3 | 14.1 | 6.04 | 2.80 |
|  |  | n=2 | 36.7 | 15.0 | 7.57 | 2.75 |
|  |  | n=3 | 33.7 | 15.8 | 7.91 | 1.38 |
| Mean (pg/mL) | | | 36.3 | 15.8 | 8.41 | 4.16 |
| SD (pg/mL) | | | 3.55 | 1.77 | 1.48 | 1.27 |
| %CV (%) | | | 9.8 | 11.2 | 17.5 | 30.5 |
| Pooled SDs (pg/mL) | | | 2.21 | | | |

Abbreviation: % CV: Coefficient of variation expressed as a percentage.

**Supplementary Table 2A.** Cross-reactivity with slow-growing NTMs

| Species | Strain | LAM  (pg/mL) |
| --- | --- | --- |
| *M. algericum* | DSM 45454 | <10.0 |
| *M. alsense* | DSM 45230 | 169,964 |
| *M. angelicum* | DSM 45057 | 295,046 |
| *M. arosiense* | DSM 45069 | 440,173 |
| *M. arupense* | DSM 44942 | <10.0 |
| *M. asiaticum* | ATCC 25276 | 160,113 |
| *M. avium* | ATCC 700898 | 427,031 |
| *M. avium* subsp. *avium* | ATCC 25291 | 302,727 |
| *M. avium* subsp. *paratuberculosis* | ATCC 19698 | 206,073 |
| *M. avium* subsp. *silvaticum* | ATCC 49884 | 296,660 |
| *M. bohemicum* | JCM 12402 | 178,660 |
| *M. botniense* | JCM 17322 | 56,393 |
| *M. branderi* | ATCC 51789 | 80,007 |
| *M. celatum* | ATCC 51131 | 133,973 |
| *M. colombiense* | JCM 16228 | 281,160 |
| *M. conspicuum* | ATCC 700090 | 171,360 |
| *M. cookii* | ATCC 49103 | 180,793 |
| *M. doricum* | JCM 12405 | <10.0 |
| *M. engbaekii* | ATCC 27353 | <10.0 |
| *M. europaeum* | DSM 45397 | 87,240 |
| *M. flavescens* | ATCC 14474 | <10.0 |
| *M. florentinum* | JCM 14740 | 299,320 |
| *M. fragae* | DSM 45731 | 274,833 |
| *M. gastri* | ATCC 15754 | 199,560 |
| *M. genavense* | ATCC 51234 | 284,480 |
| *M. gordonae* | ATCC 14470 | 230,960 |
| *M. haemophilum* | ATCC 29548 | 1,285,600 |
| *M. heckeshornense* | DSM 44428 | 64,313 |
| *M. heidelbergense* | ATCC 51253 | 122,880 |
| *M. heraklionense* | JCM 30995 | <10.0 |
| *M. hiberniae* | ATCC 49874 | <10.0 |
| *M. interjectum* | ATCC 51457 | 157,513 |
| *M. intermedium* | ATCC 51848 | 360,480 |
| *M. intracellulare* subsp. *chimaera* | JCM 14737 | 351,233 |
| *M. intracellulare* subsp. *intracellulare* | ATCC 13950 | 322,153 |
| *M. intracellulare* subsp. *yongonense* | DSM 45126 | 605,165 |
| *M. kansasii* | ATCC 12478 | 188,987 |
| *M. koreense* | DSM 45576 | <10.0 |
| *M. kubicae* | ATCC 700732 | 139,440 |
| *M. kumamotonense* | JCM 13453 | <10.0 |
| *M. kyorinense* | JCM 15038 | 359,387 |
| Species | Strain | LAM  (pg/mL) |
| *M. lacus* | JCM 15657 | 579,153 |
| *M. lentiflavum* | ATCC 51985 | 314,693 |
| *M. longobardum* | DSM 45394 | <10.0 |
| *M. malmoense* | ATCC 29571 | 211,447 |
| *M. mantenii* | JCM 18113 | 183,740 |
| *M. marinum* | ATCC 00927 | 170,973 |
| *M. marseillense* | JCM 17324 | 300,547 |
| *M. minnesotense* | JCM 17932 | <10.0 |
| *M. montefiorense* | ATCC BAA-256 | 263,288 |
| *M. nebraskense* | DSM 44803 | 23,726 |
| *M. nonchromogenicum* | ATCC 19530 | <10.0 |
| *M. noviomagense* | JCM 16367 | 63,405 |
| *M. palustre* | DSM 44572 | 178,749 |
| *M. paraense* | DSM 46749 | 270,005 |
| *M. paraffinicum* | ATCC 12670 | 259,551 |
| *M. paragordonae* | JCM 18565 | 206,933 |
| *M. parakoreense* | DSM 45575 | <10.0 |
| *M. parascrofulaceum* | JCM 13015 | 190,023 |
| *M. paraseoulense* | JCM 16952 | 308,405 |
| *M. paraterrae* | DSM 45127 | 179,282 |
| *M. parmense* | JCM 14742 | 179,608 |
| *M. persicum* | DSM 104278 | 131,930 |
| *M. pseudoshottsii* | JCM 15466 | 72,211 |
| *M. riyadhense* | DSM 45176 | 210,940 |
| *M. saskatchewanense* | JCM 13016 | 219,031 |
| *M. scrofulaceum* | ATCC 19981 | 228,902 |
| *M. senuense* | JCM 16017 | <10.0 |
| *M. seoulense* | JCM 16018 | 206,160 |
| *M. sherrisii* | DSM 45441 | 43,352 |
| *M. shigaense* | JCM 32072 | 205,790 |
| *M. shimoidei* | ATCC 27962 | 82,853 |
| *M. shinjukuense* | JCM 14233 | 892,696 |
| *M. shottsii* | JCM 12657 | 185,406 |
| *M. simiae* | ATCC 25275 | 395,378 |
| *M. stomatepiae* | JCM 17783 | 374,349 |
| *M. szulgai* | ATCC 35799 | 243,540 |
| *M. terrae* | ATCC 15755 | <10.0 |
| *M. timonense* | JCM 30726 | 315,519 |
| *M. triplex* | DSM 44626 | 366,045 |
| *M. triviale* | ATCC 23292 | <10.0 |
| *M. tusciae* | JCM 12692 | <10.0 |
| *M. ulcerans* | ATCC 19423 | 1,492 |
| *M. virginiense* | DSM 100883 | <10.0 |
| *M. vulneris* | JCM 18115 | 196,814 |
| *M. xenopi* | ATCC 19250 | 84,169 |

Concentrations above 10 pg/mL, indicating cross-reactivity, are highlighted in gray.

**Supplementary Table 2B.** Cross-reactivity with rapid-growing NTMs

| Species | Strain | LAM  (pg/mL) |
| --- | --- | --- |
| *M. abscessus* subsp. *abscessus* | ATCC 19977 | <10.0 |
| *M. abscessus* subsp. *bolletii* | JCM 15297 | <10.0 |
| *M. abscessus* subsp. *massiliense* | JCM 15300 | <10.0 |
| *M. agri* | ATCC 27406 | <10.0 |
| *M. aichiense* | ATCC 27280 | 41,642 |
| *M. alvei* | JCM 12272 | <10.0 |
| *M. anyangense* | JCM 30275 | 26,461 |
| *M. arabiense* | JCM 18538 | <10.0 |
| *M. arcueilense* | DSM 46715 | <10.0 |
| *M. aromaticivorans* | JCM 16368 | 2,587 |
| *M. aubagnense* | JCM 15296 | <10.0 |
| *M. aurum* | ATCC 23366 | <10.0 |
| *M. austroafricanum* | ATCC 33464 | <10.0 |
| *M. bacteremicum* | DSM 45578 | <10.0 |
| *M. boenickei* | JCM 15653 | <10.0 |
| *M. bourgelatii* | DSM 45746 | 39,107 |
| *M. brisbanense* | JCM 15654 | <10.0 |
| *M. brumae* | ATCC 51384 | <10.0 |
| *M. canariasense* | JCM 15298 | <10.0 |
| *M. celeriflavum* | JCM 18439 | <10.0 |
| *M. chelonae* | ATCC 35752 | <10.0 |
| *M. chitae* | ATCC 19627 | <10.0 |
| *M. chloropenolicum* | ATCC 49826 | <10.0 |
| *M. chubuense* | ATCC 27278 | <10.0 |
| *M. conceptionense* | JCM 15299 | <10.0 |
| *M. confluentis* | ATCC 49920 | <10.0 |
| *M. cosmeticum* | JCM 14739 | 21.1 |
| *M. crocinum* | JCM 16369 | 10,363 |
| *M. diernhoferi* | ATCC 19340 | <10.0 |
| *M. duvalii* | ATCC 43910 | <10.0 |
| *M. elephantis* | JCM 12406 | <10.0 |
| *M. fallax* | ATCC 35219 | <10.0 |
| *M. farcinogenes* | ATCC 35753 | <10.0 |
| *M. fluoranthenivorans* | JCM 14741 | 17.4 |
| *M. fortuitum* subsp. *acetamidolyticum* | ATCC 35931 | <10.0 |
| *M. fortuitum* subsp. *fortuitum* | ATCC 6841 | <10.0 |
| *M. franklinii* | DSM 45524 | <10.0 |
| *M. frederiksbergense* | DSM 44346 | <10.0 |
| *M. gadium* | ATCC 27726 | <10.0 |
| *M. gilvum* | ATCC 43909 | <10.0 |
| *M. goodii* | ATCC 700504 | <10.0 |
| *M. hassiacum* | JCM 12690 | <10.0 |
| *M. helvum* | JCM 30396 | <10.0 |
| *M. hippocampi* | JCM 30996 | <10.0 |
| Species | Strain | LAM  (pg/mL) |
| *M. hodleri* | JCM 12141 | <10.0 |
| *M. holsaticum* | JCM 12374 | <10.0 |
| *M. houstonense* | JCM 15656 | <10.0 |
| *M. immunogenum* | DSM 45595 | <10.0 |
| *M. insubricum* | JCM 16366 | <10.0 |
| *M. iranicum* | JCM 17461 | <10.0 |
| *M. komossense* | ATCC C33013 | 1,139 |
| *M. litorale* | JCM 17423 | <10.0 |
| *M. llatzerense* | JCM 16229 | <10.0 |
| *M. lutetiense* | DSM 46713 | <10.0 |
| *M. madagascariense* | JCM 13574 | 1,104 |
| *M. mageritense* | ATCC 700351 | <10.0 |
| *M. monacense* | JCM 15658 | <10.0 |
| *M. montmartrense* | DSM 46714 | 26.2 |
| *M. moriokaense* | ATCC 43059 | <10.0 |
| *M. mucogenicum* | ATCC 49650 | <10.0 |
| *M. murale* | JCM 13392 | <10.0 |
| *M. neoaurum* | ATCC 25795 | <10.0 |
| *M. neworleansense* | JCM 15659 | <10.0 |
| *M. novocastrense* | JCM 18114 | <10.0 |
| *M. obuense* | JCM 6372 | <10.0 |
| *M. pallens* | JCM 16370 | 4,334 |
| *M. parafortuitum* | ATCC 19686 | <10.0 |
| *M. peregrinum* | ATCC 14467 | <10.0 |
| *M. phlei* | ATCC 11758 | <10.0 |
| *M. phocaicum* | JCM 15301 | <10.0 |
| *M. porcinum* | ATCC 33776 | <10.0 |
| *M. poriferae* | ATCC 35087 | <10.0 |
| *M. psychrotolerans* | JCM 13323 | <10.0 |
| *M. pulveris* | ATCC 35154 | <10.0 |
| *M. pyrenivorans* | JCM 15927 | <10.0 |
| *M. rhodesiae* | ATCC 27024 | 13,620 |
| *M. rufum* | JCM 16372 | <10.0 |
| *M. rutilum* | JCM 16371 | <10.0 |
| *M. salmoniphilum* | DSM 43276 | <10.0 |
| *M. saopaulense* | JCM 32436 | <10.0 |
| *M. sarraceniae* | JCM 30395 | <10.0 |
| *M. sediminis* | JCM 17899 | <10.0 |
| *M. senegalense* | ATCC 35796 | <10.0 |
| *M. septicum* | ATCC 700731 | <10.0 |
| *M. setense* | JCM 15660 | 10.7 |
| *M. smegmatis* | ATCC 19420 | <10.0 |
| *M. sphagni* | DSM 44076 | <10.0 |
| *M. stephanolepidis* | JCM 31611 | <10.0 |
| *M. thermoresistibile* | ATCC 19527 | <10.0 |
| *M. tokaiense* | ATCC 27282 | <10.0 |
| *M. vaccae* | ATCC 15483 | <10.0 |
| *M. wolinskyi* | ATCC 700010 | <10.0 |

Concentrations above 10 pg/mL, indicating cross-reactivity, are highlighted in gray.

**Supplementary Table 3.** Cross-reactivity with common respiratory pathogens or microorganisms in the oral cavity

| Species | Strain | McFarland | CFU/mL  (*: cell/mL) | LAM (pg/mL) |
| --- | --- | --- | --- | --- |
| *Actinomyces naeslundii* | JCM 8349 | No.2 | 2.8×10^8^ | <10.0 |
| *Actinomyces israelii* | JCM 12964 | No.2 | 4.0×10^8^ | <10.0 |
| *Aspergillus fumigatus* | JCM 1738 | No.2 | 5.4×10^7^ | <10.0 |
| *Bacteroides fragilis* | ATCC 25285 | No.2 | 1.2×10^9^ | <10.0 |
| *Bordetella pertussis* | ATCC 9797 | No.2 | 9.6×10^8^ | <10.0 |
| *Candida albicans* | NBRC 1385 | No.2 | 1.8×10^7^ | <10.0 |
| *Chlamydophila pneumoniae* | ATCC 53592 | - | * 5.9×10^5^ | <10.0 |
| *Cryptococcus neoformans* | ATCC 32045 | No.2 | 1.2×10^7^ | <10.0 |
| *Haemophilus influenzae* | JCM 33542 | No.2 | 7.0×10^8^ | <10.0 |
| *Haemophilus parainfluenzae* | ATCC 33392 | No.2 | 1.1×10^9^ | <10.0 |
| *Klebsiella pneumoniae* | ATCC 13883 | No.2 | 9.6×10^8^ | <10.0 |
| *Legionella pneumophila* | ATCC 33152 | No.2 | 1.6×10^9^ | <10.0 |
| *Moraxella catarrhalis* | ATCC 25238 | No.2 | 1.6×10^8^ | <10.0 |
| *Mycoplasma pneumoniae* | ATCC 15531 | No.2 | 2.4×10^7^ | <10.0 |
| *Nocardia asteroides* | JCM 3384 | No.2 | 2.0×10^8^ | 323 |
|  |  | - | 1.0×10^8^ | 46.2 |
|  |  | - | 1.0×10^7^ | <10.0 |
| *Nocardia farcinica* | JCM 3088 | No.2 | 3.8×10^8^ | 30.3 |
|  |  | - | 1.0×10^8^ | <10.0 |
| *Porphyromonas gingivalis* | ATCC 33277 | No.2 | 3.0×10^8^ | <10.0 |
| *Pseudomonas aeruginosa* | JCM 5962 | No.2 | 3.4×10^8^ | <10.0 |
| *Staphylococcus aureus* | JCM 20624 | No.2 | 3.8×10^8^ | <10.0 |
| *Streptococcus agalactiae* | JCM 5671 | No.2 | 1.6×10^8^ | <10.0 |
| *Streptococcus equi* | ATCC 33398 | No.2 | 1.2×10^8^ | <10.0 |
| *Streptococcus pyogenes* | JCM 5674 | No.2 | 1.2×10^8^ | <10.0 |
| *Streptococcus anginosus* | JCM 12993 | No.2 | 6.0×10^8^ | <10.0 |
| *Streptococcus constellatus* | JCM 12994 | No.2 | 8.0×10^7^ | <10.0 |
| *Streptococcus intermedius* | JCM 12996 | No.2 | 2.0×10^7^ | <10.0 |
| *Streptococcus mutans* | ATCC 25175 | No.2 | 3.2×10^8^ | <10.0 |
| *Streptococcus mitis* | JCM 12971 | No.2 | 1.1×10^9^ | <10.0 |
| *Streptococcus oralis* | JCM 12997 | No.2 | 2.2×10^8^ | <10.0 |
| *Streptococcus pneumoniae* | ATCC 33400 | No.2 | 1.9×10^9^ | <10.0 |
| *Streptococcus salivarius* | JCM 5707 | No.2 | 1.6×10^8^ | <10.0 |
| *Streptomyces griseus* | JCM 4047 | No.2 | 1.0×10^8^ | <10.0 |
| *Tannerella forsythia* | JCM 10827 | No.2 | 1.0×10^9^ | <10.0 |
| *Treponema denticola* | JCM 8153 | No.2 | 3.8×10^7^ | <10.0 |
| *Tsukamurella paurometabola* | JCM 10117 | No.2 | 2.2×10^8^ | 18.5 |
|  |  | - | 1.0×10^8^ | <10.0 |

Concentrations above 10 pg/mL, indicating cross-reactivity, are highlighted in gray.

**Supplementary Table 4. Potential interfering substances tested.**

| Potentially interfering substance | Concentration | Potentially interfering substance | Concentration |
| --- | --- | --- | --- |
| Mucin | 1,000 μg/mL | Imipenem-cilastatin sodium | 100 μg/mL |
| Blood | 10% (v/v) | Levofloxacin | 100 μg/mL |
| Anti-TB drug |  | Povidone-iodine | 100 μg/mL |
| Isoniazid | 100 μg/mL | Betamethasone sodium phosphate fradiomycin sulfate | 100 μg/mL |
| Rifampicin | 100 μg/mL | Potassium clavulanate amoxicillin hydrate | 100 μg/mL |
| Streptomycin sulfate | 100 μg/mL | Anti-HIV drug |  |
| Ethambutol | 100 μg/mL | Lamivudine | 100 μg/mL |
| Ethionamide | 100 μg/mL | Emtricitabine | 100 μg/mL |
| Pyrazinamide | 100 μg/mL | Abacavir sulfate | 100 μg/mL |
| Kanamycin sulfate | 100 μg/mL | Tenofovir disoproxil fumarate | 100 μg/mL |
| Enviomycin sulfate | 100 μg/mL | Efavirenz | 100 μg/mL |
| Anti- pneumoniae drug |  | Etravirine | 100 μg/mL |
| Azithromycin | 100 μg/mL | Rilpivirine | 100 μg/mL |
| Clarithromycin | 100 μg/mL | Atazanavir sulfate | 100 μg/mL |
| Cefditoren pivoxil | 100 μg/mL | Maraviroc | 100 μg/mL |
| Minocycline hydrochloride | 100 μg/mL |  |  |
